# Wearing a face mask against Covid-19 results in a reduction of social distancing

**DOI:** 10.1371/journal.pone.0243023

**Published:** 2020-12-07

**Authors:** Alice Cartaud, François Quesque, Yann Coello

**Affiliations:** 1 CNRS, UMR 9193 - SCALab - Sciences Cognitives et Sciences Affectives, Univ. Lille, Lille, France; 2 Inserm, CHU Lille, U1172 - LilNCog - Lille Neuroscience & Cognition, Univ. Lille, Lille, France; National Institutes of Health, UNITED STATES

## Abstract

In the context of the Covid-19 pandemic, barrier gestures such as regular hand-washing, social distancing, and wearing a face mask are highly recommended. Critically, interpersonal distance (IPD) depends on the affective dimension of social interaction, which might be affected by the current Covid-19 context. In the present internet-based experimental study, we analyzed the preferred IPD of 457 French participants when facing human-like characters that were either wearing a face mask or displaying a neutral, happy or angry facial expression. Results showed that IPD was significantly reduced when characters were wearing a face mask, as they were perceived as more trustworthy compared to the other conditions. Importantly, IPD was even more reduced in participants infected with Covid-19 or living in low-risk areas, while it was not affected by the predicted health of the characters. These findings shed further light on the psychological factors that motivate IPD adjustments, in particular when facing a collective threat. They are also of crucial importance for policy makers as they reveal that despite the indisputable value of wearing a face mask in the current pandemic context, their use should be accompanied by an emphasis on social distancing to prevent detrimental health consequences.

## Introduction

The Covid-19 pandemic began in China in December 2019 and quickly spread around the world, with 3 889 841 cases reported in 187 countries as of May 8, 2020 (Covid-19 interactive dashboard) [1]. To slow down the pandemic, it is critical to ensure that human behavior with respect to preventing infection is represented appropriately. In accordance with WHO guidelines, many governments recommended the use of barrier gestures in social contexts such as regular hand-washing, maintaining an inter-individual distance of at least 1 meter, and wearing a medical mask [2]. Although highly encouraged due to its obvious sanitary impact, the wearing of a face mask has social consequences that have not yet been studied in depth, and its interaction with other barrier gestures such as social distancing is unknown.

Indeed, since the pioneering work of Hall [3] and Hediger [4], social interactions are known to require a fine adjustment of interpersonal distance (IPD). Selecting an appropriate IPD involves two constraints: the need to approach conspecifics given the interaction’s physical constraints and the need to maintain a margin of safety to protect the body from potential hazards [5–7]. IPD is thus not consistent across social situations, but is modulated by physical, cognitive and affective factors [8]. For instance, increasing the dimensions of conscious body representation using tools [9] produces IPD extension [10]. Likewise, IPD increases when facing conspecifics with angry compared to happy or neutral facial expressions [11–13], and is also atypical in people with socio-emotional deficits [14–16]. Importantly, interacting with people wearing a face mask might alter in the first place the affective dimension of social interactions [17–19].

Given the social context associated with Covid-19, it is essential to understand how IPD, a determining factor in blocking contamination, would be influenced by barrier gestures such as wearing a face mask, especially in view of the current and general deconfinement of populations around the world. The effects on IPD might be even harder to anticipate as quarantine periods generally lead to massive behavioral and emotional changes [20]. This is a critical issue, as a potential negative effect could be that wearing a face mask significantly enhances the feeling of safety despite the pandemic context and could jeopardize other health recommendations such as social distancing. Through a massive internet-based experimental study, we investigated this issue by asking participants to estimate whether the distance at which virtual characters were presented was appropriate or not for interacting with them. The virtual characters either wore a face mask, or wore no mask but displayed a happy, angry or neutral facial expression. The use of emotional expressions provides a well-established referential to investigate the (positive-negative) emotional effect of the face mask on IPD [11–13]. Indeed, if the presence of a face mask induces a negative feeling and is interpreted as a "threatening" cue, in particular in the current pandemic context, one should expect the IPD to increase as this is observed with characters displaying a negative emotion (angry), in comparison to the neutral condition. On the contrary, if the presence of a face mask induces a positive feeling and is interpreted as a "protective" cue, one should expect the IPD to decrease in comparison to the neutral condition, as this is the case for characters displaying a positive emotion (happy).

## Method

### Participants

Four hundred and fifty-seven adult volunteers (323 women) completed the entire experiment (*M*age = 31.53, *SD*age = 13.37). The sample size was not determined a priori as the authors expected to include as many participants as possible before the end of the Covid-19 quarantine period in France. However, the sample obtained largely exceeds the minimal sample size (n = 50) required to reasonably observe an effect characterized by a relatively small effect size (Cohen’s d = 0.4) and a standard power criterion (0.8). Written informed consent was obtained from each participant and the protocol received approval by the local institutional ethics committee (CESC Lille, Ref. 2020-425-S83).

### Apparatus and stimuli

**Fig 1:**
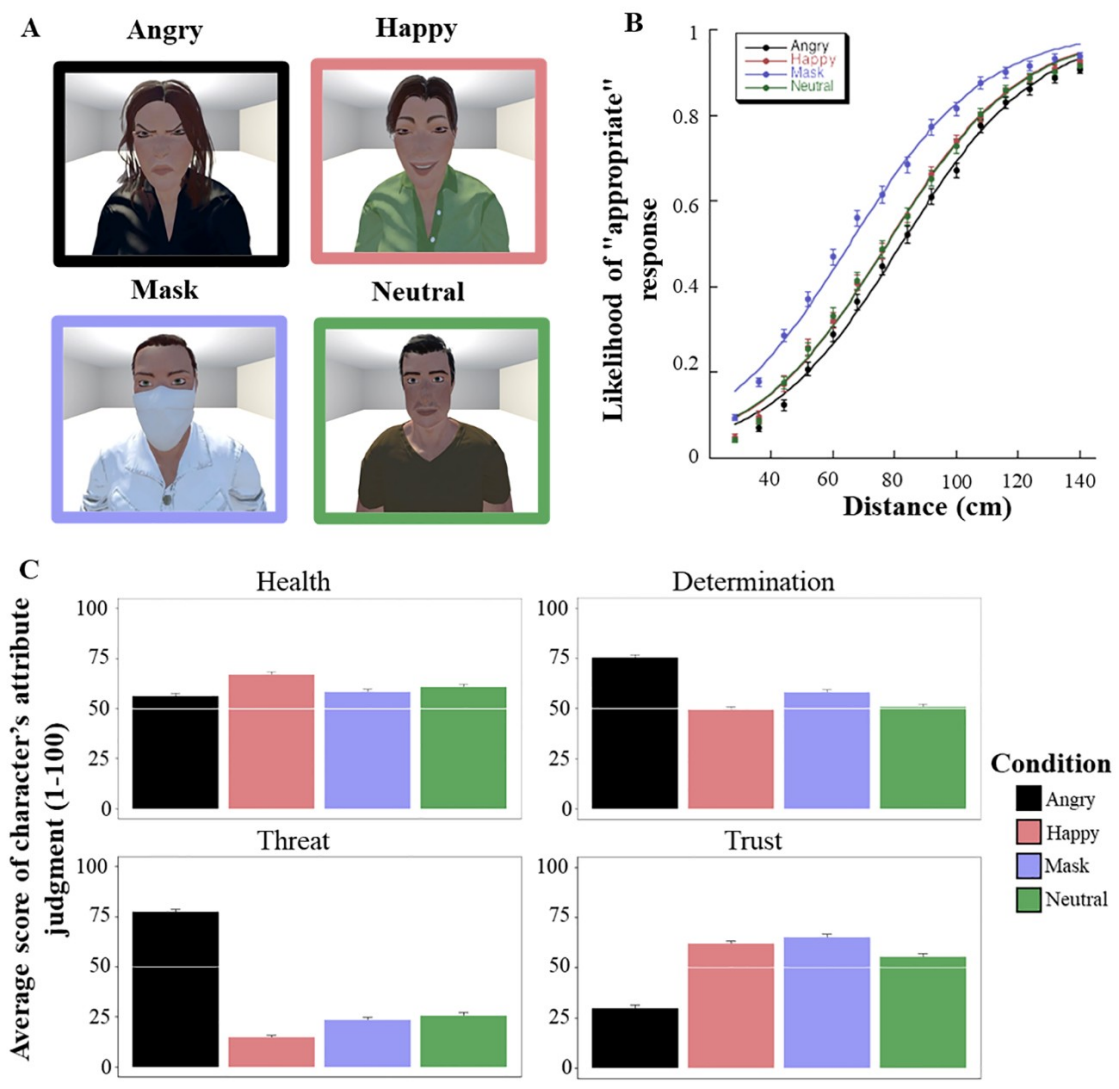
Stimuli used in the experiment and graphical representations of IPD and characters’ attributes judgments. (A) Examples of the characters used in the experiment (shown at a distance of 36 cm) in the different FE conditions. (B) Logistic regressions relating to the likelihood of “appropriate” responses as a function of the distance according to the characters FE (Angry, Happy, Mask, Neutral). (C) Mean score and 95% Confidence Interval obtained in characters’ attribute judgements (Trustworthy, Threatening, Healthy, Determined) as a function of the characters FE (Angry, Happy, Mask, Neutral).

The experiment was created on lab.js builder [21], run online, and hosted on the CNRS web server. Advertisement was shared on social and professional networks. The stimuli consisted of eight male and female virtual characters selected from the ATHOS database (all stimuli are available at: https://osf.io/sp938) [22]. A total of 4 male and 4 female characters were used in the present experiment. They were presented in an empty room with an angry, happy or neutral facial expression, or with a white face mask. When the virtual character had a face mask, the facial emotion was neutral so as to avoid confounding factors [23]. Both the characters and the empty room were built on Unity (2018.2.21f1 version). The facial expressions (FE) were randomly assigned to the characters providing thus for each participant a specific set of 4 characters with 4 FEs for each gender. The characters were presented at different distances along the virtual mid-body sagittal axis of the participants. Distances varied from 28 to 140 cm with respect to the proximal side of the virtual room (see Fig 1 for an illustration). The distance increment was 8 cm, resulting in 15 possible distances. Variables were manipulated with a within-subject design and the order of the 240 stimuli presented was fully randomized (Gender of the virtual character [2] * FE [4] * Distance [15] * Repetition [2]).

### Procedure and design

After having completed a short questionnaire concerning general information (the full questionnaire is available in S1 File), participants had to perform two tasks. The first task was to judge whether the IPD between themselves and a virtual character was appropriate for social interaction or not. The characters (female and male) were presented one by one, standing motionless at a distance ranging from 28 to 140 cm from the proximal side of the virtual room, and with different FEs (anger, happy, neutral, face mask). Each virtual character was presented twice. Responses were provided by pressing the “L” (appropriate) or “S” (inappropriate) keyboard keys (which are separated apart symmetrically on “Azerty” keyboard). Participants were instructed to respond spontaneously and as fast as possible. A 10-trial training session on independent virtual characters (not used in the following task) was administered before the experimental session to operationalize the associations between responses and keys. After 120 trials, a break was proposed to participants, if needed. The second task consisted in explicit judgements of the characters’ attributes. Participants were presented sequentially with the characters used in the first task. They were displayed at a fixed distance, 61 cm away, and several questions appeared below. The characters, presented in random order, were evaluated on whether they were “threatening”, “determined” and “trustworthy” [24] and also “healthy” due to the obvious interest given the present health context. Responses were provided by positioning a cursor on a horizontal line (100 units) with the label "really agree" on the right side and "really disagree" on the left side. Participants used their trackpad or mouse to position the cursor.

### Data analysis

The data were collected the last two weeks before the end of the French quarantine period (two weeks before 11^th^ of May 2020). Statistical analyses were carried out using generalized linear model regression (GLM) and linear models, with R (version 3.5.1) and R Studio software (version 1.1.463), for IPD and characters’ attributes respectively. Post-hoc comparisons were carried out using Bonferroni correction for the logistic regression and Tukey HSD test for the linear models (lsmeans package, version 2.30–0). The probability of “appropriate” responses (y) was analyzed using GLM (logistic regression) as a function of the Distance (28 cm to 140 cm), FE of virtual characters (mask, angry, happy, neutral), Virtual characters’ gender (female, male), Covid-19 contamination (yes, no), and Area risk level (high, low), according to the equation:

$$\begin{array}{c} y\sim \frac{1}{1+e^{-(\alpha +\sum_{i=1}^{n}\beta_{i}X_{i})}} \end{array}$$ (1)

where X_i_ corresponds to each predictor with i = 1, …, n (here n = 5, see conditions described above), α is the intercept (i.e., the coefficient of the FE used as reference, here the face mask condition), and β_i_ corresponds to the coefficient of the contrast between each predictor (X_i_) and the coefficient of the reference (statistically significant when p < .05). We also tested for interaction between FE and Covid19 contamination; FE and Virtual character’ gender; and FE and Area risk level. Given that none of these interactions was significant, they were removed from the model. Because the coefficients were not directly interpretable, they were subsequently converted into odds ratios (exp(β_i_)), which expressed the ratio between the odds of answering “appropriate” in a given condition and the odds of answering “appropriate” in a reference condition. The same model was recomputed twice, changing the FE of reference in order to obtain the coefficients of the remaining two-by-two comparisons (neutral FE vs happy and angry FE; happy FE vs angry FE).

The logistic regression parameters α and β of the within-subject conditions (FE of virtual characters, Virtual characters’ gender) reported in Table 1 were also used to estimate the boundary of appropriate IPD (as descriptive statistics), hereinafter referred to as preferred IPD. Preferred IPD corresponded to the distance at which occurred the transition between appropriate and inappropriate responses (i.e., inflection point of the logistic regression, y = 0.5). For each condition, preferred IPD corresponded to (-α + β_Condition_)/β_Distance_. Preferred IPD of the between-subject conditions (Covid-19 contamination, Area risk level) were obtained by recomputing a simplified model (only taking into account the distance) using a subset of data for each condition.

**Table 1:** Coefficients of the logistic regressions for the different variables.

Reference	Estimate	Coefficient (β)	Standard Error	z value	p	Odds ratio
α (intercept)		-3.054	0.027	-114.044	<0.001	
Distance		0.046	0.001	167.857	<0.001	1.047
Face Mask	Angry	-0.754	0.022	-34.712	<0.001	0.47
	Happy	-0.529	0.022	-24.433	<0.001	0.589
	Neutral	-0.544	0.022	-25.106	<0.001	0.581
Neutral	Happy	0.015	0.021	0.684	ns	1.015
	Angry	-0.211	0.021	-9.85	<0.001	0.81
Happy	Angry	-0.225	0.021	-10.532	<0.001	0.798
Female characters	Male characters	-0.114	0.015	-7.532	<0.001	0.892
No Covid-19	Covid-19	0.128	0.024	5.285	<0.001	1.136
Area risk level high	Area risk level low	0.185	0.017	10.545	<0.001	1.203

Odds ratios represent odds of answering “appropriate” when exposed to a Condition compared to the odds of answering “appropriate” when exposed to the Reference.

Concerning subjective evaluations of the characters’ attributes (threat, health, trust and determination), they were analyzed as a function of the characters’ FE (angry, happy, neutral, face mask), participants’ Covid-19 contamination (yes, no) and risk level of geographical area (low, high). Data and statistical analysis are available on the OSF platform (https://osf.io/utb4c).

## Results

Out of the 457 participants living in 55 different French departments, 51 declared being or having been contaminated by Covid-19 and 341 lived in a high-risk area (according to the French government’s classification). When testing how participants judged IPD, the results showed that the appropriate distance was on average 76.68 cm (Fig 1B), but that it was influenced by the characters’ FE. Preferred IPD was much shorter for the characters with a face mask (66.7 cm) than when they had a neutral (78.58 cm) FE. Interestingly, IPD was also shorter when compared to the characters with a happy (78.26 cm) or angry (83.18 cm) FE (all p<0.01). However, characters with a neutral FE had a shorter preferred IPD than characters with an angry FE (p<0.01), but not different from those with a happy FE (p>.10). Overall, these distances were modulated by individual factors. On average, preferred IPD was shorter when facing a female (75.43 cm) than male characters (77.93 cm, p < 0.001). It was also shorter when participants had been contaminated with the Covid-19 (3.2 cm, p<0.01) or when they lived in a low-risk area (3.79 cm, p <0.01). However, no interaction with the FE emerged.

Regarding character’s attributes (Fig 1C), a main effect of FE emerged for threat (F = 1453.46, p < 0.001), health (F = 41.24, p < 0.001), trust (F = 404.68, p < 0.001) and determination (F = 277.36, p < 0.01) evaluations. Characters with a face mask were evaluated as slightly more threatening (Mthreat = 23.33, CI = ± 1.51) than those with a happy FE (Mthreat = 14.63, CI = ± 1.27, p <0.001), but less than those with an angry FE (Mthreat 77.26, CI = ± 1.49, p < 0.001), and not different from those with a neutral FE (Mthreat = 25.60, CI = ± 1.57, p = 0.14). They were also evaluated as less healthy (Mhealth = 58.31, CI = ± 1.32) than those with a happy FE (Mhealth = 66.82, CI = ± 1.41, p < 0.001), but not different from those with an angry (Mhealth = 56.35, CI = ± 1.40, p = 0.20) or neutral FE (Mhealth = 60.83, CI = ± 1.41, p = 0.058). The latter were significantly different from each other (p < 0.01). Characters with a face mask were rated as more trustworthy (Mtrust = 65.1, CI = ± 1.48) than the characters with an angry FE (Mtrust = 29.73, CI = ± 1.6, p<0.01), neutral FE (Mtrust = 55.38, CI = ± 1.51, p < 0.001) or happy FE (Mtrust = 61.78, CI = ± 1.66, p = 0.02). Furthermore, they were evaluated as being more determined (Mdetermined = 58.16, CI = ± 1.38) than characters with a happy (Mdetermined = 49.28, CI = ± 1.46, p < 0.001) or neutral FE (Mdetermined = 50.79, CI = ± 1.49, p < 0.001), but less than those with an angry FE (Mdetermined = 75.54, CI = ± 1.34, p < 0.001). Finally, the evaluations of threat (F = 6.24, p = 0.01) and trust (F = 6.69, p = < 0.01) were dependent on the area risk level. Individuals living in a low-risk area rated the characters as less threatening (Mthreat = 33.6, CI = ± 2.15) than those living in a high-risk area (Mthreat = 35.76, CI = ± 1.25, p = 0.01). They also evaluated the characters as more trustworthy (Mtrust = 54.77, CI = ± 1.77) than individuals living in a high-risk area (Mtrust = 52.4, CI = ± 1.05, p = 0.01).

## Discussion

The Covid-19 pandemic represents a massive global health crisis with an unprecedented social and behavioral impact. The consistent message conveyed by health stakeholders is that the struggle against the pandemic requires significant behavioral changes. In the present study, we investigated to what extent barrier gestures interact, and in particular how wearing a face mask impacts social distancing, an essential measure against Covid-19 transmission. By using an original online paradigm in a lockdown context, our aim was to evaluate the (positive-negative) emotional valence carried by the face mask through its effect on IPD, and to compare this effect to that associated with emotional facial expressions [11–13]. We observed a significant decrease in preferred IPD when the social interaction involved a character wearing a face mask in comparison to a character with no face mask but displaying a happy, angry, or neutral facial expression. In addition to this first result, we found that area risk level regarding Covid-19 contamination affected preferred IPD. The lesser the expected risk in a particular area, the less social distancing seemed paramount to individuals. Moreover, a similar effect held for individuals contaminated with Covid-19, who felt that a shorter IPD was appropriate. One interpretation could be that being already affected by Covid-19, they might not experience the strict need to adopt barrier measures to protect themselves. Another interpretation could be that because they were more prone to select a shorter distance, they were concurrently more exposed to Covid-19 contamination. Further experiments will be needed to disentangle these two interpretations. In any case, the present findings call for high vigilance regarding social distancing policies. They deserve particular attention in the present context as threatening contexts classically lead people to seek social interactions and physical contacts [25–27]. Furthermore, since wearing a face mask cannot be considered as a sufficient safety barrier gesture in itself [28, but see 29], the present findings highlight the need to foster vigilance regarding individual practices, especially in “low-risk” areas.

Concerning subjective evaluations of the characters, those displaying an angry facial expression were rated as more threatening than the others. Interestingly, characters with happy facial expressions were evaluated as less threatening than those wearing a face mask, despite the fact that we observed smaller IPD when interacting with the latter. At first sight, these results might be surprising regarding previous findings on spatial adjustment to threatening stimuli [11–13]. However, masked, neutral and happy characters were all associated with very limited levels of threat. Moreover, the characters wearing a face mask were evaluated as more trustworthy than the others. This could have led to the reduced IPD observed with masked characters [30, 31], in relation to the positive feeling triggered by the mask, and also because “morality” judgments (as opposed to “competence” judgements) represent the core determinant of approach–avoidance tendencies toward conspecifics [32]. Therefore, perceived determination was unrelated to the regulation of IPD, as a proxy of the competence dimension. Finally, characters with a face mask were evaluated as less healthy than those with a happy facial expression, but no different from the characters with an angry or neutral facial expression. Overall, health judgements were relatively high for all characters, and were not related to the regulation of IPD. Consequently, distancing behavior based on simple visual markers might not operate in the current Covid-19 context as it is a largely “invisible” disease that remains asymptomatic in a large part of the population [33].

Critically, the present study also replicates classical findings. Specifically, we observed an increase in IPD in the presence of angry facial expressions in comparison to neutral or happy facial expressions [11–13]. In the same vein, smaller IPD were judged appropriate when interacting with female rather than male virtual characters (Cartaud et al., 2020; Iachini et al., 2016). Altogether, the fact that classical effects such as the influence of the gender of stimuli and valence of facial expressions were replicated here, underlines the good external validity of our stimuli and paradigm.

It is noteworthy that the present study aimed at quantifying the impact of emotional valence associated with face mask on social distancing in a pandemic context. This was allowed by the comparison of estimated IPD for characters wearing a face mask with conditions classically investigated (i.e., emotional faces) [11–13]. On purpose, our experimental design did not allow us to investigate the interaction between displayed emotion and the presence of a face mask as this question did not represent a critical sanitary issue. This may represent a limit of the present study. This and other specific issues will need to be addressed in future work, taking into account that a recent study demonstrated that emotional identification is strongly impaired by the presence of a face mask [23]. Furthermore, the online design of the present study did not allow us to test for a potential risk of infections on IPD adjustment. Indeed, the fact that this study was run online prevents any infection, which can have an effect on observed IPD adjustment. Future investigations involving more ecological design would thus be worth considering.

Recent works [34] highlighted the difficulty of making public policy and government decisions based solely on rationalization, as multiple cognitive biases stand in the way of risk prevention in social contexts. Among these biases leading to maladjustment of social behaviors, people generally underestimate health-related risks, find it unnatural to respect strict isolation as a means of protecting themselves and others, and have only a limited awareness of the actions that pose a health risk. Although the present study calls for generalization in more ecological settings, it provides further evidence of these biases by showing that the mere sight of a person wearing a face mask is enough to trigger a strong feeling of safety that acts against the simplest rule of social distancing. Accordingly, general recommendations to wear a face mask in society as an efficient barrier gesture against Covid-19 must be accompanied by a strong incentive to respect social distancing.

## Supporting information

S1 File (PDF)
