# Supplementary material for: Wearing a face mask against Covid-19 results in a reduction of social distancing
Source: PLoS One. 2020 Dec 7;15(12):e0243023. doi: 10.1371/journal.pone.0243023 (PMC7721169; doi:10.1371/journal.pone.0243023)
Supplement: S1 File — (PDF) [file pone.0243023.s001.pdf]

Questionnaire

- Age
- Gender (Male/Female/Other)
- Height
- Weight
- Region of residence
- At the present time, do you feel anxious (continuous scale: Not at all- extremely)
- At the present time, do you feel depressed (continuous scale: Not at all- extremely)
- What is your current situation: working at workplace/ homeworking/ suspended job/ volunteer (related to covid-19)
- Is the quarantine going to have consequences on your professional activity in the future (No/ small/ significant/ very important)
- Do you currently use public transport services (every day/ several times a week/ once a week/ once or twice a month/ never)
- Are you or have you been contaminated by Covid-19 (Yes, I was/ I am presently/there has been no diagnosis, but I think so/ there has been no diagnosis, but I do not think so/ no)
- Do you have any relative who is or has been contaminated by Covid-19 (Yes, I was/ I am presently/there has been no diagnosis, but I think so/ there has been no diagnosis, but I do not think so/ no)
- Are you presently in quarantine (yes/ no/ no but I use to be)
- Date of the beginning of your quarantine
- Date of the ending of your quarantine (if applicable)
- During the period of quarantine, have there been any significant changes in your interactions with others (yes/no)
    - Changes in frequency (increase in the number of interaction/ decrease/ no change)
    - Changes in the number of people (increase/ decrease/ no change)
    - Changes in intensity (I feel closer to the people I am interacting with/ I feel farther/ no change)
- To what extent the quarantine changed your daily organization (continuous scale : Not at all- extremely)
- To what extent do you worry for your health (continuous scale : Not at all- extremely)
- To what extent do you worry for the health of your relatives (continuous scale : Not at all- extremely)
- Do you wear a face mask (all day long/ every day, for a few hours/ several times a week/ less than once a week/ never)
- Do your relatives wear a face mask (all day long/ every day, for a few hours/ several times a week/ less than once a week/ never)
- How often do you get the latest information on covid-19 (continuously/ several times a day/ once a day/ several times a week/ less than once a week
- How do you get the latest information on covid-19 (information site on the internet/ social network/ TV/ Radio/ written press
- Do you trust media regarding covid-19 information (not at all/ rather not/ indifferent/ rather yes/ absolutely)
- Do you trust French politics regarding covid-19 (not at all/ rather not/ indifferent/ rather yes/ absolutely)

- Do you trust Science and researchers regarding covid-19 (not at all/ rather not/ indifferent/ rather yes/ absolutely)
